# SARS-CoV-2 ORF10 hijacking ubiquitination machinery reveals potential unique drug targeting sites

**DOI:** 10.1016/j.apsb.2024.05.018

**Published:** 2024-05-22

**Authors:** Kaixiang Zhu, Lili Song, Linyue Wang, Lei Hua, Ziyu Luo, Tongyun Wang, Bo Qin, Shuofeng Yuan, Xiaopan Gao, Wenyi Mi, Sheng Cui

**Affiliations:** a NHC Key Laboratory of Systems Biology of Pathogens, National Institute of Pathogen Biology, Chinese Academy of Medical Sciences and Peking Union Medical College, Beijing 100730, China; b Key Laboratory of Pathogen Infection Prevention and Control (Peking Union Medical College), Ministry of Education, Beijing 100730, China; c The Province and Ministry Co-Sponsored Collaborative Innovation Center for Medical Epigenetics, Key Laboratory of Immune Microenvironment and Disease (Ministry of Education), Tianjin Medical University General Hospital, Department of Immunology, Tianjin Medical University, Tianjin 300070, China; d Medical School, Yan'an University, Shaanxi Province, Yan'an 716000, China; e State Key Laboratory of Emerging Infectious Diseases, Li Ka Shing Faculty of Medicine, the University of Hong Kong, Pokfulam, Hong Kong SAR, China; f Department of Microbiology, Li Ka Shing, Faculty of Medicine, the University of Hong Kong, Pokfulam, Hong Kong SAR, China

**Keywords:** SARS-CoV-2, ORF10, Crystal structure, cullin-2 RING E3 ligase, Drug design

## Abstract

Viruses often manipulate ubiquitination pathways to facilitate their replication and pathogenesis. CUL2^ZYG11B^ known as the substrate receptor of cullin-2 RING E3 ligase, is bound by SARS-CoV-2 ORF10 to increase its E3 ligase activity, leading to degradation of IFT46, a protein component of the intraflagellar transport (IFT) complex B. This results in dysfunctional cilia, which explains certain symptoms that are specific to COVID-19. However, the precise molecular mechanism of how ORF10 recognizes CUL2^ZYG11B^ remains unknown. Here, we determined the crystal structure of CUL2^ZYG11B^ complexed with the N-terminal extension (NTE) of SARS-CoV-2 ORF10 (2.9 Å). The structure reveals that the ORF10 N-terminal heptapeptide (NTH) mimics the Gly/N-degron to bind CUL2^ZYG11B^. Mutagenesis studies identified key residues within ORF10 that are key players in its interaction with CUL2^ZYG11B^ both in ITC assay and *in vivo* cells. In addition, we prove that enhancement of CUL2^ZYG11B^ activity for IFT46 degradation by which ORF10-mediated correlates with the binding affinity between ORF10 and CUL2^ZYG11B^. Finally, we used a Global Protein Stability system to show that the NTH of ORF10 mimics the Gly/N-degron motif, thereby binding competitively to CUL2^ZYG11B^ and inhibiting the degradation of target substrates bearing the Gly/N-degron motif. Overall, this study sheds light on how SARS-CoV-2 ORF10 exploits the ubiquitination machinery for proteasomal degradation, and offers valuable insights for optimizing PROTAC-based drug design based on NTH CUL2^ZYG11B^ interaction, while pinpointing a promising target for the development of treatments for COVID-19.

## Graphical abstract

This paper studied a mechanism of SARS-CoV-2 ORF10 binding to CUL2^ZYG11B^ by mimicking Gly/N-degrons, revealing potential unique drug-targeting sites.

**Figure:**
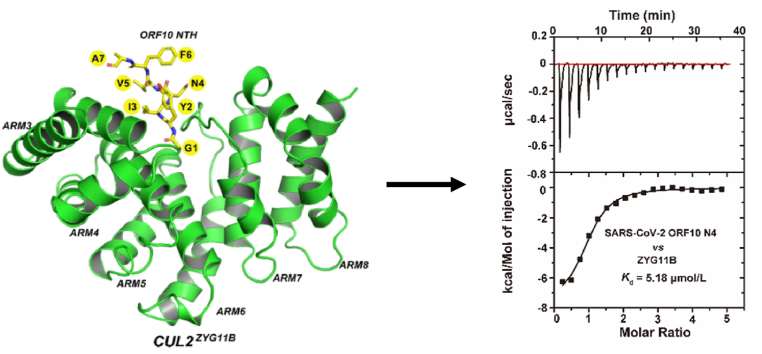

## 1 Introduction

The signs of COVID-19 include coughing, fever, muscle pain, exhaustion, and a diminished ability to taste and smell^1^^,^^2^. SARS-CoV-2 predominantly infects ciliated cells and impairs the function of the cilia on the cell surface^3^, ^4^, ^5^, which causes anosmia and ageusia^6^. Previous studies demonstrate that cilia dysfunction is induced by SARS-CoV-2 ORF10, resulting in ubiquitination-dependent proteasomal degradation of multiple ciliary proteins^7^^,^^8^, which may explain some of the symptoms that are specific to COVID-19.

The SARS-CoV-2 ORF10 protein, a speculative viral protein consisting of 38 amino acids, is produced by the accessory region of the genome located at the 3′ end. It is exclusive to SARS-CoV-2^9^, ^10^, ^11^. The absence of any similarity in sequence between the SARS-CoV-2 ORF10 and other identified coronavirus proteins implies that it could potentially be responsible for the unique pathogenesis and/or symptoms linked to COVID-19. The function of SARS-CoV-2 ORF10 remains controversial. A systematic interactome analysis of all SARS-CoV-2 encoded proteins established a link between the ORF10 protein and CUL2^ZYG11B^, a component of the CUL2 RING E3 ligase complex^12^. Nevertheless, a subsequent study confirmed that the interaction between ORF10 and CUL2^ZYG11B^ is dispensable for the E3 activity of CUL2^ZYG11B^ and does not impact SARS-CoV-2 infection *in vitro*^13^. By contrast, other studies suggest that ORF10 plays a significant role in immune evasion^14^, ^15^, ^16^, ^17^. A recent study reinvestigated the function of ORF10 systematically and proposed that interaction between ORF10 and CUL2^ZYG11B^ leads to significant enhancement of the E3 ligase activity of CUL2^ZYG11B^, this increases ubiquitin-dependent degradation of IFT46, thereby impairing biogenesis and maintenance of cilia^7^^,^^8^. These data provide a compelling pathological explanation for some COVID-19-specific symptoms with a phenotype related to dysfunction of cilia^7^^,^^8^.

CUL2^ZYG11B^, a member of the CRL2 or CUL2 complex, controls protein degradation by selectively directing N-terminal glycine degrons, also known as Gly/N-degrons, for ubiquitination and subsequent proteasomal degradation^18^^,^^19^. Protein ubiquitination is a critical driver of infection; Therefore, many viruses have developed diverse strategies to hijack the ubiquitin/proteasome system to induce degradation of host antiviral proteins and increase viral pathogenesis^20^^,^^21^. The crystal structures of various Gly/N-degrons-CUL2^ZYG11B^ complexes reveal the N-terminal glycine, as well as a bulky or aromatic residue following glycine are key residues preferred by CUL2^ZYG11B^ ^22^. Intriguingly, the beginning part of SARS-CoV-2 ORF10 N-terminus is initiated by methionine, glycine, and tyrosine residues, with the methionine being consistently eliminated by methionine aminopeptidases^23^; this process presumably makes the N-terminus of ORF10 recognizable by CUL2^ZYG11B^ because it mimics the Gly/N-degron. Therefore, it is probable that ORF10 binds to the substrate binding site of CUL2^ZYG11B^ to inhibit CUL2^ZYG11B^ mediated-target protein degradation by blocking binding to substrates harboring the Gly/N-degrons. However, the molecular mechanism by which CUL2^ZYG11B^ recognizes ORF10 to enhance its activity for IFT46 degradation, and whether ORF10 functions as a Gly/N-degron mimic to interfere with the ability of CUL2^ZYG11B^ to target substrates bearing Gly/N-degrons, remains unclear.

In this study, we determined the crystal structure of CUL2^ZYG11B^ complexed with the N-terminal extension (NTE) of SARS-CoV-2 ORF10 to 2.9 Å resolution. Analysis of this structure revealed that the ORF10 N terminal heptapeptide (NTH) mimics the Gly/N-degron by recognizing CUL2^ZYG11B^. We also conducted an isothermal titration calorimetric (ITC) assay *in vitro*, as well as cell-based experiments*,* which identified key ORF10 residues as being important for the interaction with CUL2^ZYG11B^. Furthermore, we demonstrate that ORF10-mediated enhancement of CUL2^ZYG11B^ activity for IFT46 degradation correlates with the affinity of ORF10 for CUL2^ZYG11B^. Finally, we used a Global Protein Stability (GPS) assay to demonstrate that the NTE of ORF10 acts as a Gly/N-degron mimic to bind CUL2^ZYG11B^, thereby competitively inhibiting the binding of CUL2^ZYG11B^ to its target substrate. In summary, the data reveal the molecular mechanism underlying hijacking of ubiquitination activity by ORF10 to promote proteasomal degradation and identify a candidate for the development of targeted treatments for COVID-19.

## 2 Materials and methods

### 2.1 Reagents and cells

A rabbit antibody specific for HA (1:2000, 3724S) was purchased from Cell Signaling Technology. A rabbit antibody specific for Flag (1:2000, ab205606) was purchased from Abcam. Anti-Flag and anti-alpha tubulin antibodies were purchased from Proteintech. A mouse antibody specific for Myc (1:5000, Cat#05-724-25UG) was purchased from MERCK. HRP Goat Anti-Rabbit IgG (H+L), HRP Goat Anti-Mouse IgG (H+L), and a mouse antibody specific for GAPDH were purchased from ABclonal. Anti-Flag Magnetic Agarose (Thermo Fisher Scientific: A36797) was purchased from Thermo Fisher Scientific.

### 2.2 Design of the plasmid constructs

The coding frames of human CUL2^ZYG11B^, human IFT46, and SARS-CoV-2 ORF10 were chemically synthesized in Genscript (Supporting Information Table S1). PCR was used to amplify CUL2^ZYG11B^ (residues 490–728) with the primer pairs mentioned in Supporting Information Table S2. The product was cloned into a pET-28a-6×His-SUMO vector harboring a TEV-cleavable site (pET28a-6×His-SUMO-TEV-CUL2^ZYG11B(490–728)^) for expression as previously described^24^^,^^25^. The pHA-CUL2^ZYG11B^, pMyc-IFT46, pFlag-ORF10 and pFlag-ORF10 fused luciferase plasmids were constructed based on pCDNA3.1 vector with C-terminal HA, MYC, or Flag tag (Table S2).

### 2.3 Protein expression and purification

For expression and purification of the CUL2^ZYG11B(490–728)^ recombinant protein, the pET28a-6 × His-SUMO-TEV-CUL2^ZYG11B(490–728)^ plasmid was introduced into competent cells of *E.coli*, followed by an overnight incubation at 37 °C in Luria broth. For large-scale cultures, 10 mL of a culture that had been incubated overnight was employed to inoculate 1 L medium. After 3–4 h incubation, 0.5 mmol/L IPTG was introduced to induce the protein expression, followed by incubation overnight at 18 °C. The overnight cultures were gathered through centrifugation at 4000×*g* for a duration of 10 min. Subsequently, the cell pellet was resuspended in lysis buffer. The pellet was processed by ultrasonication. The supernatant containing the soluble recombinant protein was processed by loading onto Ni-NTA column. Following a wash in buffer, the desired protein was released with elution buffer. Afterwards, the SUMO-His-tag was cleaved by TEV protease. After cleavage, the flowthrough containing CUL2^ZYG11B(490–728)^ protein was gathered and filtered through a HiTrap Q HP column. The flowthrough fraction was pooled and subjected to gel filtration chromatography for further purification.

### 2.4 Crystal production and structure determination

To form co-crystals of CUL2^ZYG11B^ with the ORF10 peptide, ORF10 peptides were added to the CUL2^ZYG11B^ protein (molar ratio of 1:4). The complex was then concentrated to ∼5 mg/mL, and crystallized by combing 1 μL protein and 1 μL reservoir buffer at 20 °C. The crystals began to appear after 1 week and grew to an optimal size within 2 weeks. All diffraction data sets were conducted and collected on beamline BL10U2 and BL19U1 at the Shanghai synchrotron radiation facility (SSRF). The data were processed using the software XDS package^26^; The molecular replacement method with Phaser MR was used to solve the structure by the searcher model of the CUL2^ZYG11B^ (PDB id:7EP1)^27^. The structure was modified by Coot manually, and further refined using PHENIX^28^. Supporting Information Table S3 summarizes the data collection statistics, and structure refinement parameters.

### 2.5 Gel filtration assay

After purification, the CUL2^ZYG11B^ proteins were injected into a Superdex 200 column pre-calibrated with a standards marker. SDS-PAGE and followed Coomassie Brilliant Blue staining were used to analyze the peak fractions.

### 2.6 Isothermal titration calorimetry (ITC)

For ITC experiments, a MicroCal iTC200 calorimeter was utilized as previously described^29^, ^30^, ^31^. The CUL2^ZYG11B^ protein and the ORF10 peptides were dissolved in the same solution. The syringe was filled with the peptides (0.5–1 mmol/L), and the cell was filled with CUL2^ZYG11B^ protein (0.03 mmol/L). The peptides were titrated against proteins with a reference power of 8 (μCal/s), and 18 consecutive 2 μL injections were monitored at a stirring speed of 400 rpm. Origin was used to conduct data analysis. Each ITC experiment was repeated 3 times.

### 2.7 Co-immunoprecipitation(Co-IP) and Western blotting(WB)

Co-IP assays were conducted following the previously stated protocol^32^. Briefly, HEK293T cells in culture were transfected with the specified plasmids using Lipofectamine2000 (Thermo Fisher Scientific). Following 30 h incubation, cells were harvested and subjected to lysis for 20 min in RIPA Lysis Buffer supplemented with 1 mmol/L phenylmethanesulfonyl fluoride. The supernatant obtained was allowed to interact with anti-FLAG-magnetic beads overnight at 4 °C. Subsequently, the beads underwent five washes with wash buffer at 4 °C. The resuspended beads were then heated in loading buffer and subjected to WB analysis using antibodies.

### 2.8 Cycloheximide chase (CHX) assay

After 24 h of transfection with the specified plasmids, *de novo* protein synthesis was inhibited by the addition of 100 μg/mL cycloheximide (CHX) (MedChemExpress, HY-12320). Total protein samples were harvested at the specified time (0, 2, 4, and 6 h) after the addition of CHX, followed by Western blot analysis.

### 2.9 GPS assay

The 12-mer peptide from the beginning of SNX11 N-terminus was constructed into the pCDH-Ub-MCS-GFP-P2A-RFP vector as previously described^22^^,^^33^ (Table S2). First, stable GPS reporter cells were produced by lentivirus packaging and selected using blasticidine. Then, wild-type ORF10 was overexpressed in GPS reporter cells. Finally, the stability of the peptide-GFP conjugate was assessed by measuring the GFP and RFP ratio by using flow cytometry with RFP serving as an internal control. Flow cytometry analysis was performed using an ACEA NovoCyte instrument, and the data were analyzed using FlowJo. WB was used to examine the expression of ORF10 in GPS reporter cells.

### 2.10 Cell culture and lentiviral transduction

To generate stable GPS-reporter cells, lentiviruses were packed by co-transfecting three plasmids, the GPS reporter vector, the pMD2.G plasmid, and the psPAX2 vectors in HEK293T cells. After 48 h, infection was conducted by using collected lentiviral supernatants. Briefly, the cells were incubated with produced lentiviral supernatants plus 8 μg/mL polybrene for 48 h, then the infected cells were selected with 10 μg/mL blasticidin to produce stable GPS-reporter cells.

### 2.11 ZYG11B/ZER1 KO cells

The ZYG11B/ZER1 KO cells were generated using the pLentiCRISPRv2 system (catalog number 52961, Addgene)^34^. Briefly, three plasmids named pMD2.G, psPAX2, and lentiCRISPRv2 carrying single guide RNAs (sgRNAs) targeting ZYG11B and ZER1 were co-transfected into HEK293T cells. Table S2 lists the sgRNA sequences used. Lentiviral supernatants were collected after 2 days and used to infect cells. The lentiviral supernatants with polybrene (8 μg/mL) were incubated with GPS-reporter cells for 48 h. Finally, the cells were exposed to 2 μg/mL puromycin to select lentiCRISPR-transduced GPS-reporter cells.

### 2.12 Data availability

The accession codes: 7YC2 have been deposited in the PDB with the associated atomic coordinates and structure factors.

## 3 Results and discussion

**Figure 1:**
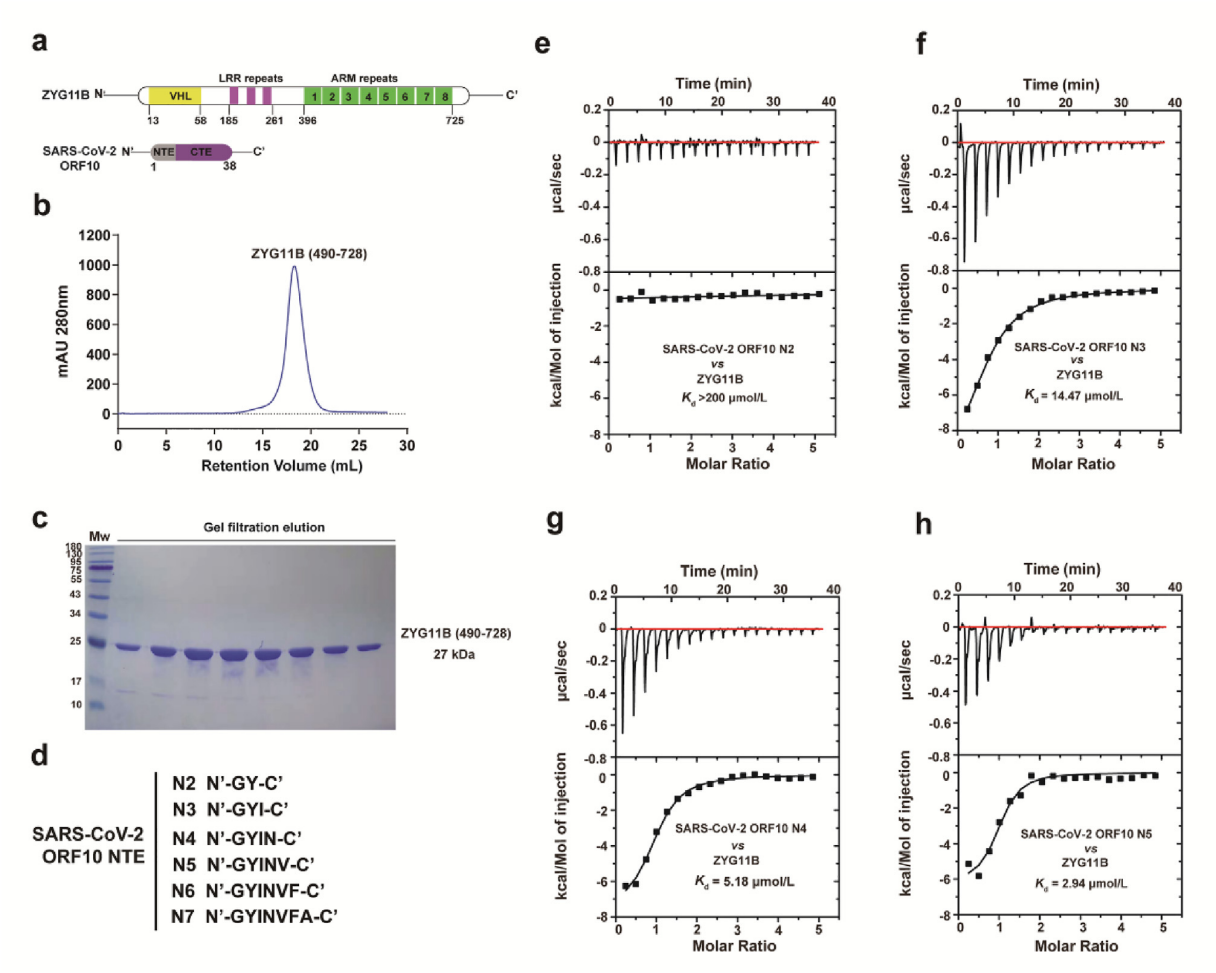
Peptides including the N-terminal extension of the ORF10 protein interact with the CUL2^ZYG11B^ with nanomolar affinity. (a) Diagram displaying the domain organization of CUL2^ZYG11B^ and the ORF10 proteins with annotations. (b) Size-exclusion chromatography profile of final purification of CUL2^ZYG11B^. (c) SDS-PAGE analysis of the eluate shown in panel b. (d) Sequence of ORF10 NTE peptides (N2–N7). (e–h) ITC titrations between SARS-CoV-2 ORF10 N2–N5 peptides and CUL2^ZYG11B^.

To gain a deeper comprehension of SARS-CoV-2 ORF10-mediated regulation of CUL2^ZYG11B^, we determined the crystal structure of the ORF10-CUL2^ZYG11B^ complex. First, we cloned, expressed, and purified recombinant CUL2^ZYG11B^ proteins containing ARM3–ARM8 repeats (residues 490–728) (Fig. 1a–c), which are necessary for recognition of the Gly/N degrons^22^. However, ectopic expression of the ORF10 protein alone, or co-expression of ORF10 with CUL2^ZYG11B^ in prokaryotic and/or eukaryotic systems was unsuccessful, most likely due to ORF10-mediated cellular cytotoxicity, as well as its small size and the potential existence of a transmembrane domain at the NTE^11^, which probably jeopardized protein expression. To get around this, we synthesized several peptides, ranging from two to seven residues in length, which are present within the ORF10 NTE. We then examined their binding affinity for CUL2^ZYG11B^ using ITC (Fig. 1d, Supporting Information Tables S4 and S5). The six and seven-residue peptides have poor solubility, and were therefore not suitable for ITC experiments; However, the remaining four peptides named N2–N5 were highly soluble, and so were used for evaluation. ITC assays revealed that a two-residue peptide named N2 did not bind to CUL2^ZYG11B^ (Fig. 1e); However, a three-residue peptide named N3 exhibited relatively strong binding affinity (*K*_d_ = 14.47 μmol/L, Fig. 1f), and the four-residue peptide N4 showed even higher binding affinity (2.7-fold higher; *K*_d_ = 5.18 μmol/L, Fig. 1g). Furthermore, the five-residue peptide N5 had a binding affinity 4.9-fold higher (*K*_d_ = 2.94 μmol/L, Fig. 1h). Thus, the binding data demonstrate that the first three residues of ORF10 are primarily responsible for its interaction with CUL2^ZYG11B^, and that its binding affinity is comparable with that of the GIMAP5 and ZNF701 Gly/N-degrons^22^ (*K*_d_ = 2.5–3.3 μmol/L). Next, we co-crystallized CUL2^ZYG11B^ along with each of these six peptides (N2–N7) (Fig. 1d) and obtained SARS-CoV-2 ORF10 N7-CUL2^ZYG11B^ crystals. We then used molecular replacement to determine the crystal structure (searching model PDB id: 7EP1). Table S3 summarizes the crystallographic statistics, as well as the parameters used for data collection and model refinement.

**Figure 2:**
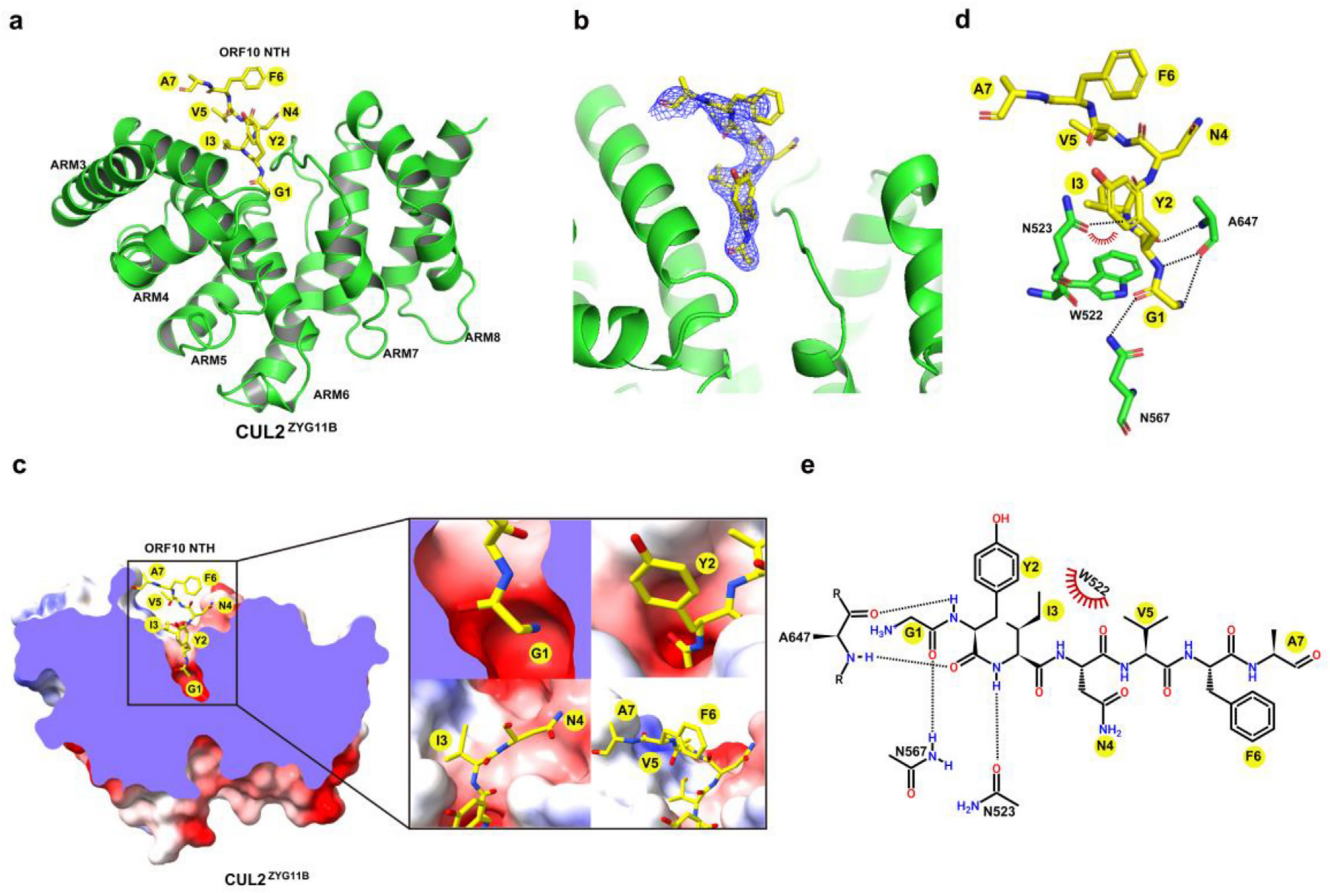
Crystal structure of ORF10 NTH complexed with CUL2^ZYG11B^. (a) The overall structure of the SARS-CoV-2 ORF10 NTH complexed with CUL2^ZYG11B^. The NTH is shown as yellow sticks, and ARM3–ARM8 is shown as a green ribbon. (b) The composite omit map for ORF10 NTH is superimposed on the model, which is denoted by blue meshes. (c) Left, cutaway view showing NTH (yellow sticks) in the Gly/N-degron binding pocket of CUL2^ZYG11B^, CUL2^ZYG11B^ is shown in a surface electrostatic potential plot. Right, magnified view of the first seven ORF10 residues of the Gly/N degron pocket of CUL2^ZYG11B^. (d) Detailed interactions between CUL2^ZYG11B^ (green stick model) and the ORF10 NTH (yellow stick model). (e) The interaction diagram showing recognition of the NTH by CUL2^ZYG11B^. The black dashed lines and red eyelashes were used to display intermolecular hydrogen bonds and hydrophobic interactions, respectively.

The crystal structure revealed that the ORF10 NTH bearing the Gly/N-like degron is positioned in the middle of the ARM3–ARM8 pattern motif within CUL2^ZYG11B^ (Fig. 2a). The electron density maps calculated by composite omit maps clearly delineate seven residues in the ORF10 NTH (Fig. 2b). The NTH of SARS-CoV-2 ORF10 possesses an inverted L-shape, which inserts into the cavity of CUL2^ZYG11B^. In particular, the first glycine (G1) is buried deep in a negatively charged binding pocket within CUL2^ZYG11B^ (Fig. 2c). The second tyrosine (Y2) is also inserted into the binding pocket through the main chain, while its side chain extends towards the outer edge of the groove (Fig. 2c). The third isoleucine (I3) and fourth asparagine (N4) residues reside at the entrance to the binding cavity, whereas the remaining three residues [valine (V5), phenylalanine (F6), and alanine (A7)] are more exposed to solvent and are expected to sit outside the binding cavity (Fig. 2c). It is probable that these three residues are less important for binding to CUL2^ZYG11B^, which agrees with the ITC assay results (Fig. 1e–h). Thus, the first three-residues peptide is sufficient to retain strong binding to CUL2^ZYG11B^, although longer peptides with four or five residues have a marginally higher binding affinity. Binding involves interactions with atoms that form the backbone of the first three residues, as well as the protonated NH_3_^+^ group of the first residue (Fig. 2d and e). Recognition of the G1 residue of the NTH appears to be particularly important. The *α*-carbonyl group of first G residue forms hydrogen bonds with the N567 side chain in CUL2^ZYG11B^, and the *α*-amino group of first G residue forms another hydrogen bond with A647 of CUL2^ZYG11B^ (Fig. 2d and e). In addition, A647 contributes to formation of two hydrogen bonds with the backbone carbonyl and amide of Y2 (Fig. 2d and e). I3, with its backbone amide, forms a single hydrogen bond with N523, and simultaneously engages in hydrophobic interactions with W522 in CUL2^ZYG11B^ (Fig. 2d and e). The remaining remnants are exposed to solvent and therefore are not engaged in particular interaction with CUL2^ZYG11B^ (Fig. 2d and e). We also used AlphaFold2 to predict the structure of full-length ORF10 in complex with CUL2^ZYG11B^. The results revealed that the full-length ORF10 adopts a disordered and coiled conformation, with its N-terminal extension positioned within the Gly/N-degron binding pocket of CUL2^ZYG11B^, mirroring closely the resolved ORF10 NTH/CUL2^ZYG11B^ structure. Superimposition of the complex structure of the full-length ORF10/CUL2^ZYG11B^ predicted by AlphaFold2 with the crystal structure of ORF10 NTH/CUL2^ZYG11B^ gave an overall rmsd value of 0.714 Å (Supporting Information Fig. S1). Overall, the recognition of the SARS-CoV-2 ORF10 NTH to CUL2^ZYG11B^ follows the Gly/N-degron as observed previously^22^.

**Figure 3:**
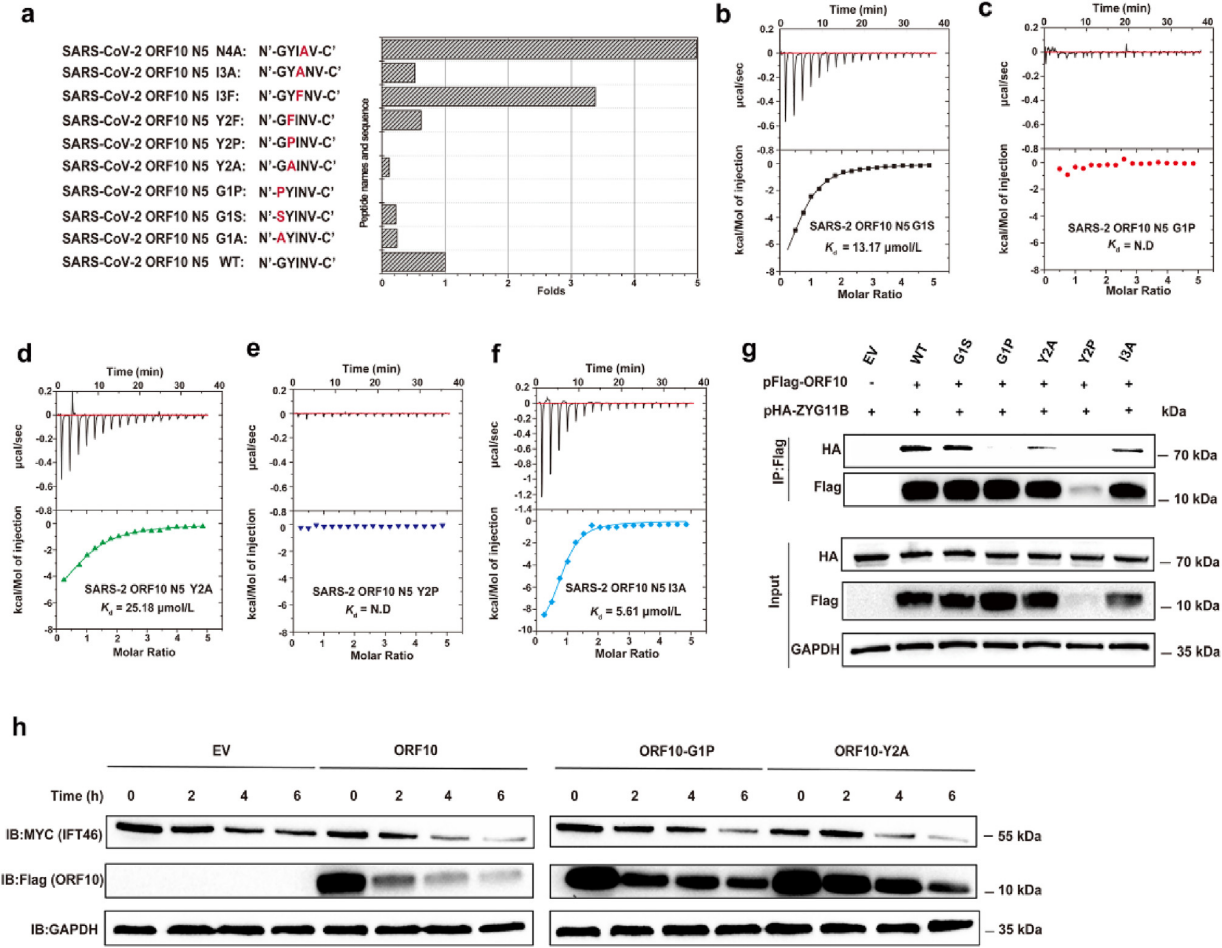
Investigation of the binding affinity of ORF10 and its mutants to CUL2^ZYG11B^, and their effect on IFT46 degradation. (a) A histogram is presented to illustrate the binding affinity of various peptides derived from the ORF10 NTE to CUL2^ZYG11B^. The *x*-axis represents the fold change (folds of binding affinity) as compared to the affinity of ORF10 N5 peptide (*K*_d_ = 2.94 μmol/L), while the *y*-axis displays different peptides names and sequences. Thus, 2.94 μmol/L/*K*_d_ of the given peptide was used to calculate the folds of binding affinity. (b–f) ITC assay demonstrating the interaction of each peptide with CUL2^ZYG11B^. (g) After co-transfection of HEK-293T cells with the specified plasmids, 24 h later, co-immunoprecipitation (Co-IP) analysis was performed to examine the interaction between CUL2^ZYG11B^ and ORF10 wild-type (WT) or mutant proteins. (h) The effects of ORF10 WT or mutant viral proteins on IFT46 degradation. Cells were co-transfected with IFT46, ORF10 WT, mutant ORF10, or empty vector (EV). A CHX assay of IFT46 was carried out. Samples were collected at 0, 2, 4, and 6 h following the administration of CHX. Immunoblotting with anti-MYC and -Flag antibodies revealed the protein levels of IFT46 and ORF10 protein. GAPDH was used as a loading control.

To ascertain the function of specific residues in the SARS-CoV-2 ORF10 NTH region in its interaction with CUL2^ZYG11B^, we created several altered peptides derived from SARS-CoV-2 ORF10 N5 and assessed their binding affinity to CUL2^ZYG11B^ using ITC assay (Tables S4 and S5). G1 fits tightly into the binding pocket of CUL2^ZYG11B^ (Fig. 2c). Mutations of the first glycine (G1A and G1S) moderately decreased the binding ability for CUL2^ZYG11B^ by ∼4 folds (Fig. 3a and b, Supporting Information Fig. S2a), when the G1P mutation was introduced, the binding affinity was abolished (Fig. 3a and c). This may be because the specific pyrrolidine ring is too large to fit into the binding pocket of CUL2^ZYG11B^. Furthermore, the Y2A mutation reduced the binding affinity by ∼8-fold (*K*_d_ = 25.18 μmol/L, Fig. 3d), and the Y2P mutation disrupted binding completely (Fig. 3e). Conversely, the Y2F mutation had little effect on binding affinity to CUL2^ZYG11B^ (*K*_d_ = 4.73 μmol/L, Supporting Information Fig. S2b). From a structural perspective, Y2 inserts into the cavity of CUL2^ZYG11B^ *via* back-bone atoms, and its side chain extends into the rim of the channel (Fig. 2c). The aromatic ring may forms an extra *π‒π* interaction with W522. As a result, the bulky aromatic residues at position 2 (Y/F) bind preferentially to CUL2^ZYG11B^, whereas replacement of these aromatic residues with a smaller residue (A), or the specific pyrrolidine ring residue (P), is strongly disfavored at this position. I3 protrudes outwards from the pocket and is coordinated by a backbone-mediated hydrogen bond with N523, and a side chain-mediated hydrophobic interaction with W522 (Fig. 2d and e). The ITC data showed that substitution of I with F increases the binding ability by ∼3 folds (*K*_d_ = 0.87 μmol/L, Fig. S2c) because the F residue may further enhance the hydrophobic interaction with W522, whereas substitution of I with A leads to a marginally reduced affinity (*K*_d_ = 5.61 μmol/L, Fig. 3f), which is likely due to loss of the hydrophobic interaction between A and W522. N4 fully extends outwards from the binding groove and is not expected to interact with CUL2^ZYG11B^. However, ITC revealed that the affinity of N4A for CUL2^ZYG11B^ is 5-fold higher (*K*_d_ = 0.59 μmol/L, Fig. S2d). This is most likely because substitution with A removes the steric hindrance created by the N residue. Taken together, the above ITC assays suggest that the first three N-terminal residues of NTH are critical for the recognition of CUL2^ZYG11B^, with the first two residues (G and Y) being the key determinant residues of the interaction.

To further confirm the structural and biophysical characterization of the ORF10-derived peptides, a co-immunoprecipitation (Co-IP) experiment was performed to study the interaction of intact ORF10 with CUL2^ZYG11B^ in cells. As expected, the Co-IP results revealed that intact ORF10 interacted with CUL2^ZYG11B^, whereas introducing mutations in SARS-CoV-2 ORF10, reduced its binding affinity to CUL2^ZYG11B^ to different degrees (Fig. 3g). This finding is consistent with our ITC results. G1P completely disrupted the binding of ORF10 to CUL2^ZYG11B^. Therefore, this key glycine is also essential for the interaction within cells. The Y2A mutation greatly impaired the binding of SARS-CoV-2 ORF10 to CUL2^ZYG11B^ (Fig. 3g). In addition, the Y2P mutant was expressed very poorly because protein folding was severely disrupted (Fig. 3g).

It has previously shown that ORF10 inhibited cilium maintenance and biogenesis by enhancing IFT46 degradation through improving the activity of CUL2^ZYG11B^ ^7^^,^^8^. Therefore, we next investigated the interactions involved in the ORF10-mediated IFT46 degradation *via* its interaction with CUL2^ZYG11B^. Cycloheximide chase (CHX) assays showed that overexpressing ORF10 led to a marked increase in IFT46 degradation compared with the negative control (Fig. 3h), which is consistent with previous results^7^. Mutations in ORF10 that reduced its binding affinity to CUL2^ZYG11B^ (as shown by the ITC assay and Co-IP assay) also damaged its ability to promote IFT46 degradation to different extents (Fig. 3h). Of note, ORF10 mutation G1P nearly abolished its ability in promoting IFT46 degradation, and ORF10 mutation Y2A elicited moderate effects on promoting IFT46 degradation. Collectively, these findings offer strong experimental evidence that ORF10-mediated degradation of IFT46 is dependent on the binding of ORF10 to CUL2^ZYG11B^. Abolishing this interaction impairs the function of ORF10.

**Figure 4:**
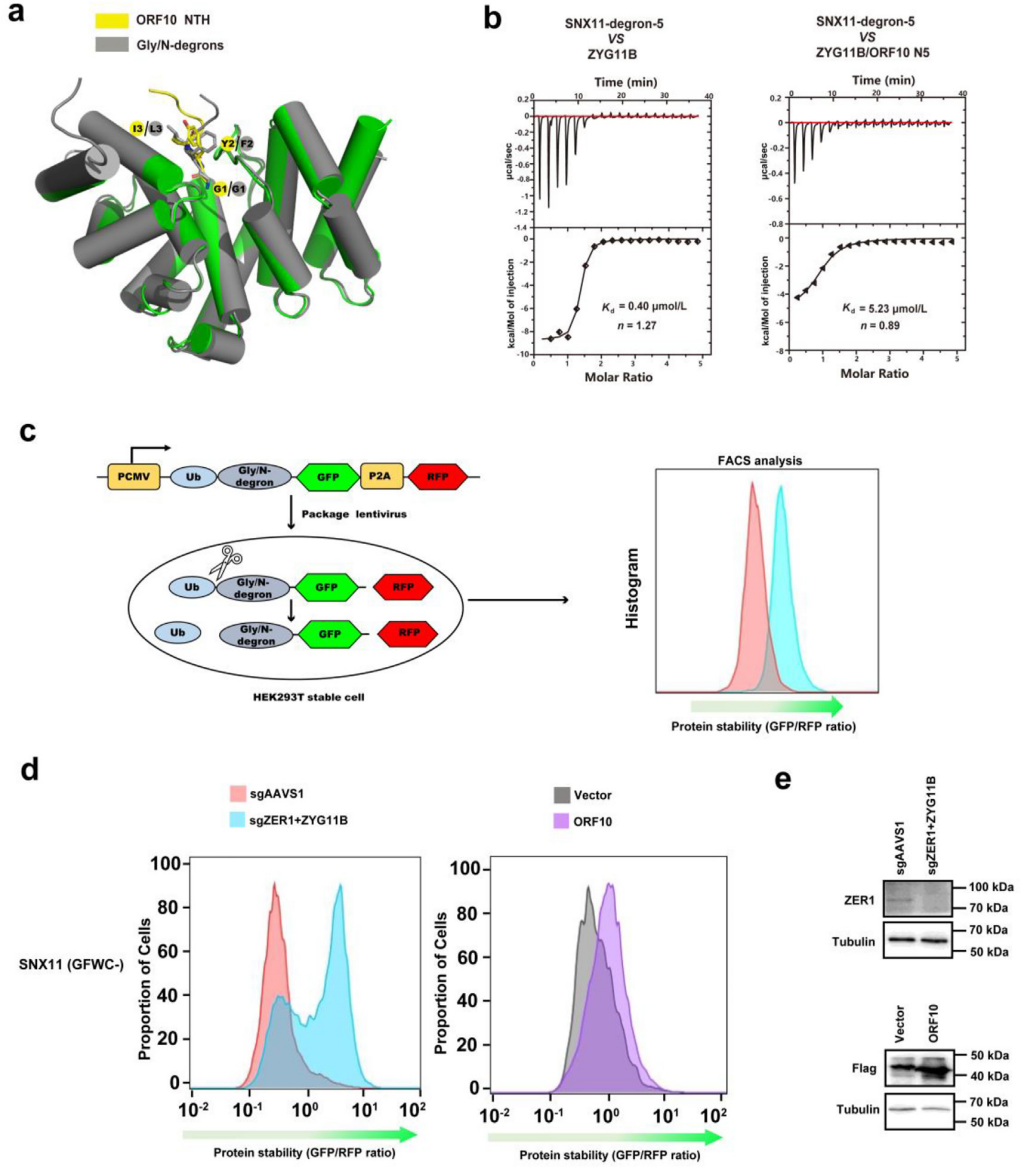
ORF10 binding to CUL2^ZYG11B^ inhibits degradation of substrates bearing the Gly/N-degron. (a) Superposition analysis of ORF10 NTH with the GFLH Gly/N-degron (PDB ID:7EP1). ORF10 NTH is shown in yellow, and GFLH Gly/N-degron are shown in gray. The first three residues are labeled. (b) Left, Original binding curves for the interaction of SNX11 degron peptide and ZYG11B; right, binding curves for pre-formed ZYG11B/ORF10 N5 peptide complex and SNX11 degron peptide. (c) The flowchart of the GPS assay. PCMV, human cytomegalovirus promoter; Ub, ubiquitin; P2A, self-cleaving 2A peptide from porcine teschovirus-1; GFP, green fluorescent protein; RFP, red fluorescent protein. (d) Left: Stability of SNX11 degron-GFP after simultaneous knockout of ZER1/ZYG11B in HEK293T cells. Right: stability of SNX11 degron-GFP upon overexpression of ORF10-fused luciferase in HEK293T cells. The GFP/RFP ratio was calculated by flow cytometry. (e) Upper, WB analysis of ZER1 expression in ZYG11B/ZER1 knock out GPS-reporter cell lines. Lower, WB analysis of ORF10 fused luciferase expression in SNX11 degron GPS-reporter cell lines.

The NTH of ORF10 binds to CUL2^ZYG11B^ in the same way as the Gly/N-degron. This suggests that the ORF10 NTH likely prevents binding to substrates containing the Gly/N-degron, thereby inhibiting the degradation of CUL2^ZYG11B^ target proteins. Superimposition of the ORF10 NTH-CUL2^ZYG11B^ complex onto the GFLH‒CUL2^ZYG11B^ complex (PDB ID:7EP1) showed that the first three residues have conserved backbone structures, and that the amino acids are very similar (Fig. 4a). To investigate the effect of the ORF10 NTH on blocking binding of the SNX11-degron to CUL2^ZYG11B^, we titrated the SNX11-degron peptide with the ORF10 NTH/CUL2^ZYG11B^complex (Tables S4 and S5). Briefly, the ORF10 NTH was pre-incubated with the CUL2^ZYG11B^ to allow complex formation, followed by the addition of the SNX11-degron peptide. The results showed that the affinity of the SNX11-degron for the ORF10 NTH-CUL2^ZYG11B^complex was ∼13 folds lower than that of the SNX11-degron peptide for CUL2^ZYG11B^ alone (Fig. 4b). To clarify whether ORF10 binds competitively to CUL2^ZYG11B^ by mimicking the Gly/N-degron, thereby preventing degradation of the substrate *in vivo*, we employed GPS to analyze their impact on the stability of SNX11-degron-fused GFP as previously described^22^^,^^33^(Fig. 4c). In this system, we initially evaluated the stability of SNX11-degron-fused GFP by measuring the GFP/RFP ratio in ZYG11B and ZER1 double knock-out (KO) cells. We found that ZYG11B and ZER1 double KO significantly increased the GFP/RFP ratio by attenuating the degradation of the SNX11-GFP chimeric protein (Fig. 4d and e). Similarly, overexpression of exogenous ORF10-fused luciferase inhibited degradation of the SNX11-GFP chimeric protein in HEK293T cells (Fig. 4d and e). Taken together, these results confirm our hypothesis that OFR10 mimics the Gly/N-degron by binding competitively to CUL2^ZYG11B^, thereby inhibiting the degradation of substrates bearing the Gly/N-degron.

**Figure 5:**
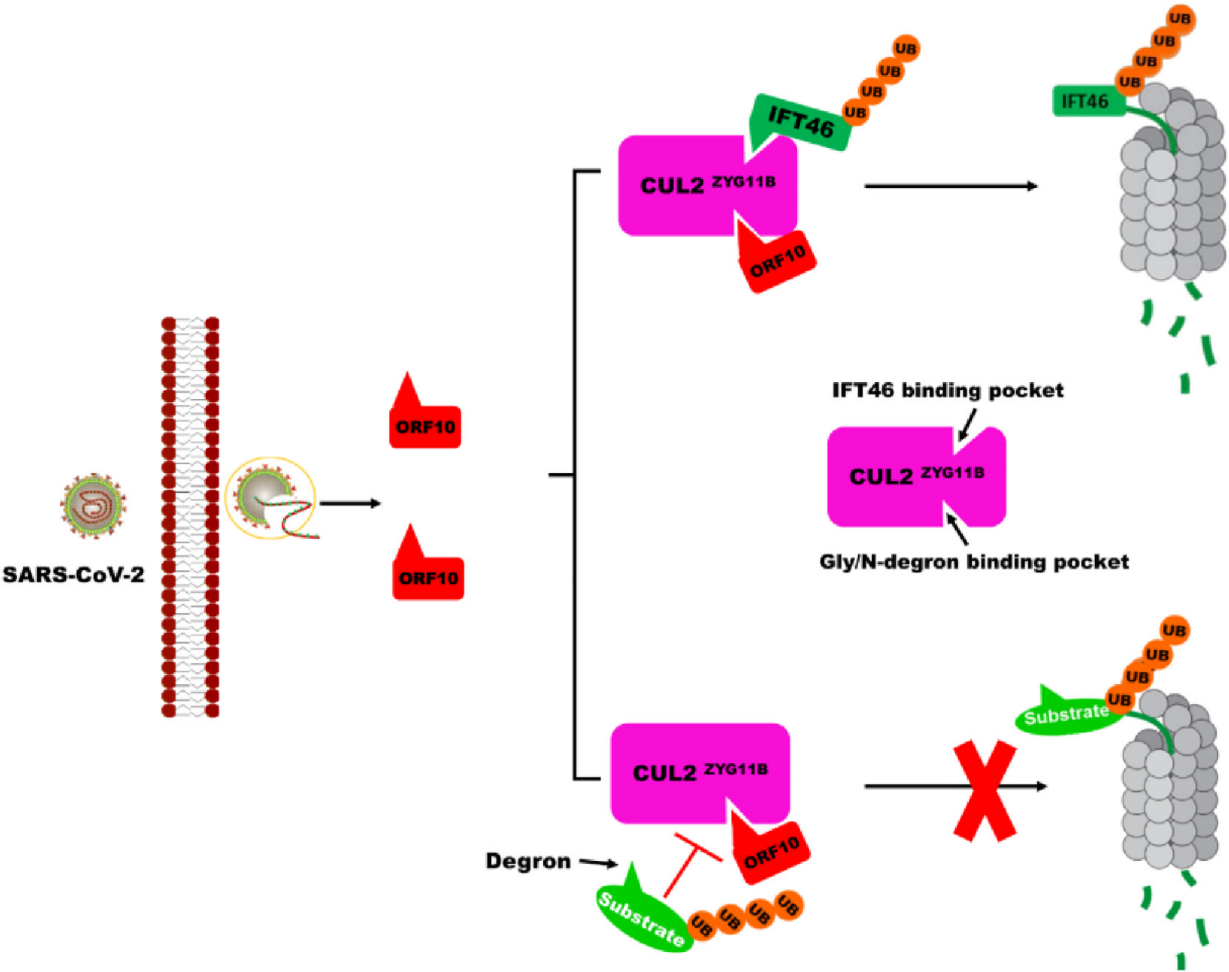
Working model showing the effect of ORF10 on the stability of CUL2^ZYG11B^ target proteins. Upon infection by SARS-CoV-2, ORF10 binds to ZYG11B to exert dual functions. On the one hand, ORF10 increases the ubiquitination activity of ZYG11B, leading to degradation of IFT46. On the other, the ORF10 N-terminal extension (NTE) acts as a Gly/N-degron mimic to bind ZYG11B and competitively inhibits binding of ZYG11B to its target substrate bearing the Gly/N-degron.

In summary, the interaction between ORF10 and CUL2^ZYG11B^ to promote IFT46 degradation and inhibit the degradation of CUL2^ZYG11B^ target substrate bearing Gly/N-degron through two different mechanisms (Fig. 5). The non-classical mechanism involves recognition of CUL2^ZYG11B^ by ORF10 *via* the Gly/N-degron pocket and of IFT46 *via* the IFT46 C2 binding pocket; ORF10 binding to the Gly/N-degron pocket in CUL2^ZYG11B^ increases its ability to degrade IFT46. The canonical way involves binding of ORF10 to the Gly/N-degron pocket of CUL2^ZYG11B^ by mimicking the Gly/N-degron; this enables to interfere with the degradation of substrates bearing the Gly/N-degron.

## Author contributions

Kaixiang Zhu: Project administration, Writing – review & editing, Writing – original draft. Lili Song: Methodology, Validation. Linyue Wang: Data curation, Software. Lei Hua: Methodology. Ziyu Luo: Methodology. Tongyun Wang: Methodology. Bo Qin: Data curation, Investigation. Shuofeng Yuan: Methodology. Xiaopan Gao: Conceptualization, Writing – original draft. Wenyi Mi: Conceptualization, Supervision. Sheng Cui: Supervision, Writing – original draft.

## Conflicts of interest

The authors declare no conflicts of interest.

## Appendix A Supplementary information

The following is the Supporting information to this article:
