# Supplementary material for: SARS-CoV-2 ORF10 hijacking ubiquitination machinery reveals potential unique drug targeting sites
Source: Acta Pharm Sin B. 2024 May 22;14(9):4164–73. doi: 10.1016/j.apsb.2024.05.018 (PMC11413682; doi:10.1016/j.apsb.2024.05.018)
Supplement: Multimedia component 1 [file mmc1.pdf]

Supporting Information for

Short communication

SARS-CoV-2 ORF10 hijacking ubiquitination machinery reveals potential unique drug targeting sites

Kaixiang Zhu[a,b,†], Lili Song[c,†], Linyue Wang[a,b,†], Lei Hua[a,d,†], Ziyu Luo[a,b], Tongyun Wang[e,f], Bo Qin[a,b], Shuofeng Yuan[e,f], Xiaopan Gao[a,b,*], Wenyi Mi[c,*], Sheng Cui[a,b,*]

[a]*NHC Key Laboratory of Systems Biology of Pathogens, National Institute of Pathogen Biology, Chinese Academy of Medical Sciences and Peking Union Medical College, Beijing 100730, China*

[b]*Key Laboratory of Pathogen Infection Prevention and Control (Peking Union Medical College), Ministry of Education, Beijing 100730, China*

[c]*The Province and Ministry Co-sponsored Collaborative Innovation Center for Medical Epigenetics, Key Laboratory of Immune Microenvironment and Disease (Ministry of Education), Tianjin Medical University General Hospital, Department of Immunology, Tianjin Medical University, Tianjin 300070, China*

[d]*Medical School, Yan'an University, Shaanxi Province, Yan'an 716000, China*

[e]*State Key Laboratory of Emerging Infectious Diseases, Li Ka Shing Faculty of Medicine, the University of Hong Kong, Pokfulam, Hong Kong SAR, China*

[f]*Department of Microbiology, Li Ka Shing, Faculty of Medicine, the University of Hong Kong, Pokfulam, Hong Kong SAR, China*

Received 21 January 2024; received in revised form 15 April 2024; accepted 9 May 2024

[†]These authors made equal contributions to this work.

[*]Corresponding authors.

E-mail addresses: panda888@ipbcams.ac.cn (Xiaopan Gao), wenyi.mi@tmu.edu.cn (Wenyi Mi), cui.sheng@ipb.pumc.edu.cn (Sheng Cui).

Supporting Figures

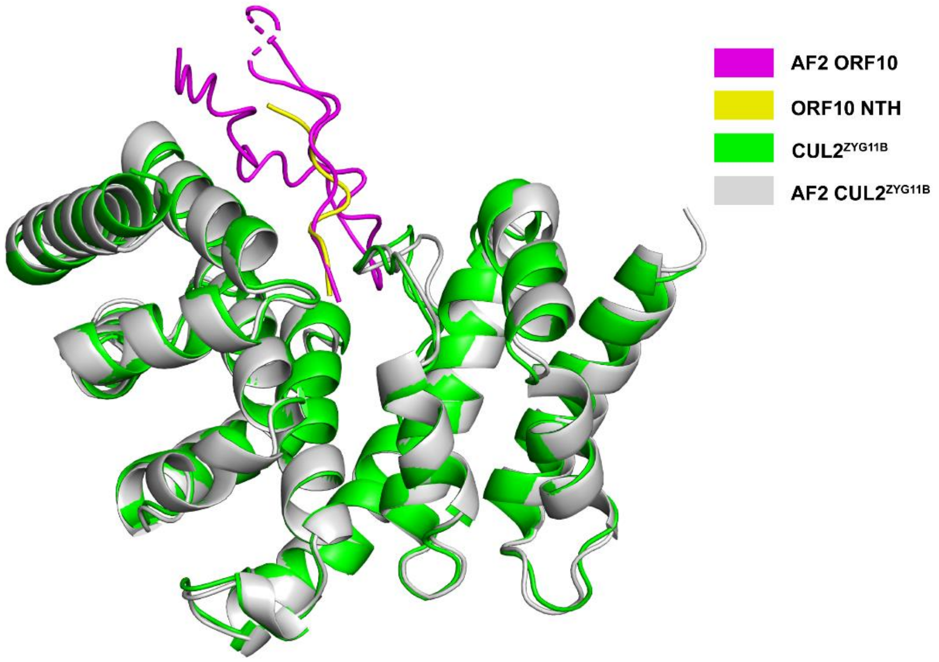

Figure S1. Superimposition of the complex structure of the full-length ORF10/CUL2^{ZYG11B} predicted by AlphaFold2 with the crystal structure of ORF10 NTH/CUL2^{ZYG11B}. In the Alphafold2 structure, full-length ORF10 is colored magenta, CUL^{ZYG11B} is colored gray. In the crystal structure, ORF10 NTH is colored yellow, CUL^{ZYG11B} is colored green.

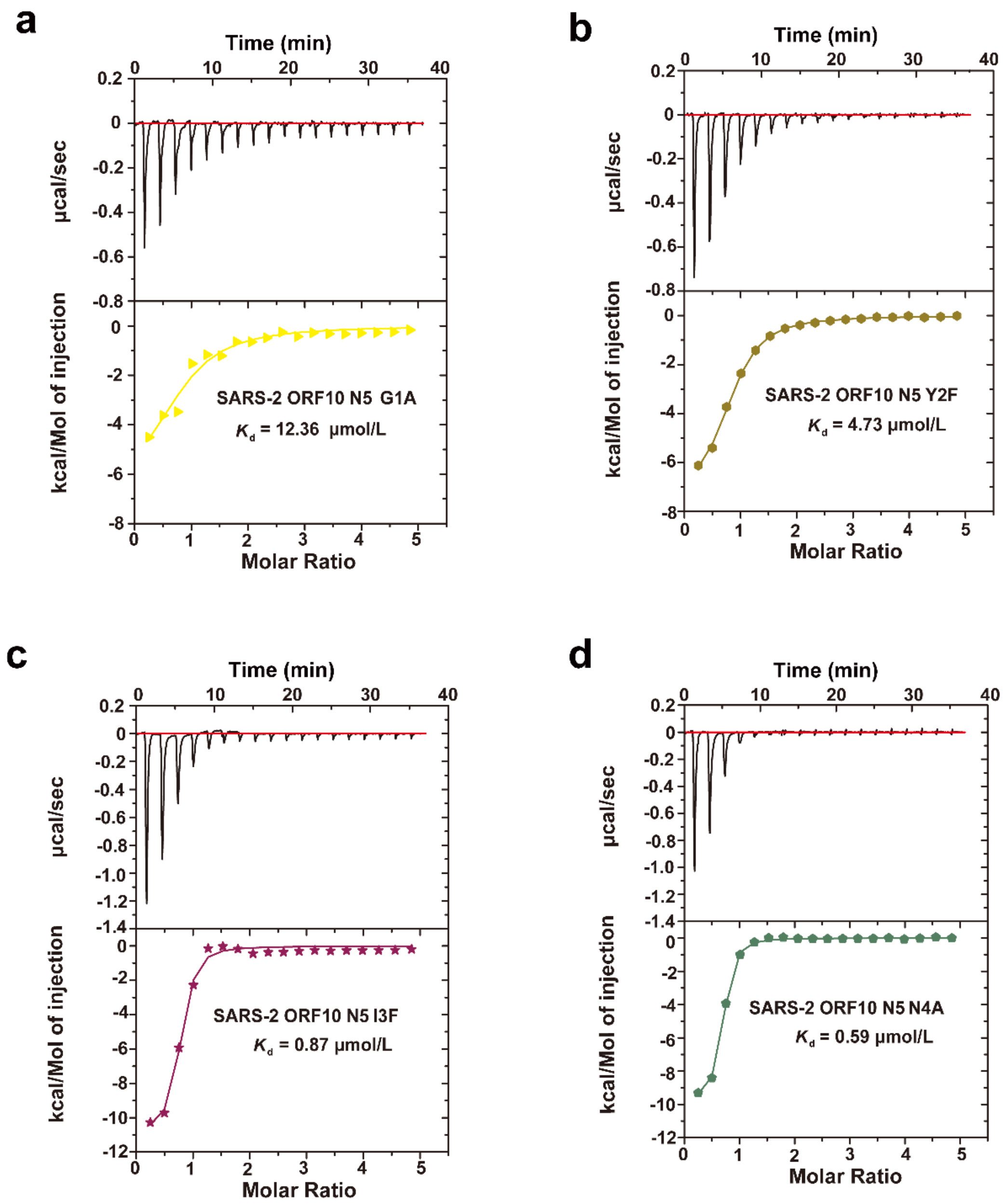

Figure S2. ITC assay between ORF10 mutants peptide and CUL2^{ZYG11B}

a-d. Binding isotherm for the interaction between ORF10 mutants peptide and CUL2^{ZYG11B}. The calculated K_d values are indicated.

Supporting Tables

Table S1. Optimized genes in this study

Gene names	Optimized genes sequence
ORF10-3xFlag	>ATGGGCTACATCAACGTGTTCGCCTTTCCTTTCACCATCTACTC<br>TCTGCTGCTGTGCAGAATGAACAGCCGGAACTACATCGCCCAG<br>GTGGACGTGGTCAATTTCAACCTGACAGACTACAAAGACGACG<br>ACGATAAGGACTACAAGGACGACGACGACAAGGATTATAAGGA<br>CGATGATGATAAGTGA
CUL2$^{ZYG11B(490\text{-}728)}$	>GAATTGTTCATCGTGCGCCAGCTGCTGCAGATCGTTAAACAGA<br>AAACCAATCAAAACAGCGTTGATACCACGCTCAAGTTCACCTT<br>GAGCGCACTGTGGAATCTGACCGACGAGTCCCCGACGACGTGC<br>CGTCATTTTATCGAGAACCAGGGCCTGGAGCTGTTCATGCGTGT<br>GCTGGAGTCGTTCCCGACTGAGTCCAGCATTCAGCAAAAGGTG<br>CTGGGCTTGTTGAACAACATCGCCGAAGTTCAAGAGTTGCACT<br>CGGAATTAATGTGGAAGGACTTCATCGATCATATCTCATCCCTGC<br>TTCACTCTGTCGAGGTGGAAGTCAGCTATTTCGCTGCGGGTATT<br>ATTGCGCATCTGATTAGCCGTGGTGAACAAGCGTGGACCCTCTC<br>TCGCTCTCAGCGTAATAGCCTGTTAGATGATCTGCACTCTGCGAT<br>TCTTAAGTGGCCTACCCCGGAATGTGAAATGGTTGCGTATCGTA<br>GCTTTAACCCGTTTTTTCCGCTGTTGGGCTGCTTTACCACTCCAG<br>GTGTGCAGCTGTGGGCGGTTTGGGCAATGCAGCACGTTTGCAG<br>CAAAAACCCGAGCCGCTACTGCAGCATGCTGATTGAGGAAGGT<br>GGTCTGCAACACCTGTACAACATTAAAGACCACGAGCACACCG<br>ACCCGCATGTGCAGCAAATCGCCGTGGCCATCTTGGACAGCCT<br>GGAGAAGCACATTGTACGC
IFT46	>ATGGCTGATAATTCAAGTGACGAATGTGAGGAGGAGAACAAC<br>AAAGTCCTGCGTGAAGGCATGCCGCAGGCACCGGGTCATAGAG<br>GCAAGGATATGGATCCGGTGCCGCCTGCTCCGGCGAGCTTGAA<br>GTGCCACCAGACCCCATCCCATGTTCTCGAGCGCGTGGGTTGGT<br>ATCGTGAATCCCAAAAACACCGCAAAGAGAAAAAGAAGACCT<br>CCCAGCTGACTCCGCAACGTGGTTTTAGCGAAAATGAAGATGA<br>CGACGACGACGATGACGACAGCAGCGAAACCGATTCCGACTCG<br>GATGACGACGATGAAGAGCACGGCGCACCGCTGGAAGGCGCG<br>TATGATCCGGCGGATTATGAACACCTGCCGGTTTCCGCGGAAAT<br>CAAAGAACTCTTCCAGTACATCTCTCGTTACACGCCGCAACTGA<br>TTGACCTGGACCACAAACTGAAACCGTTTATCCCGGATTTTATT<br>CCGGCAGTTGGTGACATCGACGCGTTCTTGAAGGTGCCGCGTC<br>CGGATGGTAAGCCGGATAATTTAGGTCTGCTGGTACTGGACGAA<br>CCGAGCACCAAACAGAGCGACCCGACGGTGTTGTCCCTGTGGT<br>TGACCGAAAACAGTAAGCAACACAACATTACCCAGCACATGAA<br>AGTGAAATCCTTGGAGGACGCTGAGAAAAACCCGAAAGCGATC

	GACACCTGGATTGAAAGCATTAGCGAGTTGCACCGCTCTAAGC CACCGGCTACCGTTCATTACACCCGTCCGATGCCTGATATCGATA CCCTGATGCAAGAATGGTCACCGGAGTTCGAGGAGCTGCTGGG CAAGGTTTCGTTGCCAACCGCTGAGATTGACTGTAGCCTTGCCG AGTACATCGACATGATTTGCGCCATCCTGGATATCCCGGTCTACA AAAGCCGTATTCAGTCTTTGCATCTGCTTTTCTCTCTGTATAGCG AGTTCAAGAACAGCCAACATTTTAAGGCGCTGGCGGAGGGCAA AAAGGCGTTCACCCCGAGCAGCAATAGCACCAGCCAGGCCGGT GATATGGAAACTCTGACGTTTTCG

Table S2. List of primers used in this study

Name	Forward primer(5'-3')	Reverse primer(5'-3')
pET28a-6His-SUMO-TEV-CUL2$^{ZYG11B(490-728)}$	CAGATTGGTGGATCCGAAAATCTATACTTTCAAAGCGAATTGTTCATCGTGCGCCAGC	TGGTGGTGGTGCTCGAGTTAGCGTACAATGTGCTTCTCC
pCDNA3.1-IFT46-MYC	TCCAGTGTGGTGGAATTCGCCACCATGGCTGATAATTCAAGTGACG	GGGCCCTCTAGACTCGAGTTACAGGTCCTCCTCACTGATCAATTTCTGCTCCGAAAACGTCAGAGTTTCCAT
pCDNA3.1-CUL2^{ZYG11B}-HA	TCCAGTGTGGTGGAATTCGCCACCATGCCCGAGGACCAGGCCGG	GGGCCCTCTAGACTCGAGTTAAGCGTAGTCTGGGACGTCGTATGGGTAGTTCAGTCTAGCCTGAGGCTGC
pCDNA3.1-SARS-CoV-2-ORF10-Flag WT	TAGTCCAGTGTGGTGGAATTCGCCACCATGGGCTACATCAACGTGTTCG	GCCCTCTAGACTCGAGCGGCCGCTCACTTATCATCATCGTCCTTATAATCC
pCDNA3.1-SARS-CoV-2-ORF10-Flag G1S	CACCATGAGCTACATCAACGTGTTCGCCTTTC	TGATGTAGCTCATGGTGGCGAATTCCACCACA
pCDNA3.1-SARS-CoV-2-ORF10-Flag G1P	CACCATGCCCTACATCAACGTGTTCGCCTTTCC	TGATGTAGGGCATGGTGGCGAATTCCACCACA
pCDNA3.1-SARS-CoV-2-ORF10-Flag Y2A	ATGGGCGCCATCAACGTGTTCGCCTTTCCTTT	ACGTTGATGGCGCCCATGGTGGCGAATTCCAC
pCDNA3.1-SARS-CoV-2-ORF10-Flag Y2P	ATGGGCCCCATCAACGTGTTCGCCTTTCCTTT	ACGTTGATGGGGCCCATGGTGGCGAATTCCAC

pCDNA3.1-SARS-CoV-2-ORF10-Flag I3A	GGCTACGCCAACGTGTTCGCCTTTCCTTTCAC	AACACGTTGGCGTAGCCCATGGTGGCGAATTC
pLentiCRISPRv2-ZYG11B-sgRNA2	CACCGAAGCTCGAAGGCCAGAAAGC	AAACGCTTTCTGGCCTTCGAGCTTC
pLentiCRISPRv2-ZER1-sgRNA1	CACCGTATGAGGAGGAGAACCCAGG	AAACCCTGGGTTCTCCTCCTCATAC
pCDH-Ub-MCS-GFP-P2A-RFP-GFWC-	CGTCTCAGAGGTGGTGGCTTTTGGTGTAGGATGTCGGAGAACCAAGAACAGGAGGAGGTGATTACAGTGCGTGCCACCAGCGCCCTG	CAGGGCGCTGGTGGCACGCACTGTAATCACCTCCTCCTGTTCTTGGTTCTCCGACATCCTACACCAAAAGCCACCACCTCTGAGACG
pcDNA3.1-ORF10-(GGGGS)2-luciferase-Flag	TGGTCAATTTCAACCTGACAGGTGGCGGTGGCTCGGGTGGCGGTGGCTCGGAAGACGCCAAAAACATAAAGAAAG	TCGTCGTCGTCTTTGTAGTCCACGGCGATCTTTCCGCC

Table S3. Data collection and refinement statistics

	SARS-CoV-2 ORF10 in complexed CUL2^{ZYG11B} (PDB ID: 7YC2)
**Data collection**	
Space group	C121
Cell dimensions	
a, b, c (Å)	106.20, 72.80,127.50
α, β, γ (°)	90.00,113.40, 90.00
X ray source	
Wavelength (Å)	0.978557
Data range (Å)	39.65-2.89
Reflections unique	38376[a]
R_{sym} [b] (last shell)	0.279 (2.157)
$I / \sigma I$ (last shell)	4.13 (0.73)
Completeness (%) (last shell)	98.4 (98.2)
Redundancy (last shell)	3.36 (3.19)
**Refinement**	
Resolution range (Å)	17.05-2.90
Reflections, cut-off, % reflections in cross validation	19869[a], F>1.35, 4.94
R_{work} [c]/ R_{free} [d] (last shell)	0.2498/0.2623 (0.3652/0.3880)

Atoms	
Non-hydrogen protein atoms	7885
Protein	7885
Solvent	0
B-factors average (Å^2)	74.22
Protein (Å^2)	74.22
Ligands (Å^2)	0
Solvent (Å^2)	0
**r.m.s.d**	
Bond lengths (Å)	0.007
Bond angles (°)	1.093
% residues in favored regions, allowed regions, outliers in Ramachandran plot	97.40,2.49,0.11

Values in parentheses are for the highest-resolution shell.

[a]Friedel pairs are treated as different reflections

[b] $R_{sym} = \sum_{hkl}\sum_j |I_{hkl,j} - I_{hkl}|/\sum_{hkl}\sum_j I_{hkl,j}$, where I_{hkl} is the average of symmetry-related observations of a unique reflection

[c] $R_{work} = \sum_{hkl} ||F_{obs}(hkl)|-|F_{calc}(hkl)||/\sum_{hkl}|F_{obs}(hkl)|$.

[d] R_{free}= the cross-validation R factor for 5% of reflections against which the model was not refined.

Table S4. Thermodynamic parameters of the binding between ORF10 synthetic peptides and CUL2^{ZYG11B}

Binding partners	***N***	***K_a, M^{-1}***	***K_d, μM***	***ΔH, cal/mole***	***ΔS, cal/mole/deg***
SARS-2 ORF10 N2 vs CUL2^{ZYG11B}	5.05	$1.35*10^3±2.81*10^4$	>200	$-2333±7.516*10^4$	6.52
SARS-2 ORF10 N3 vs CUL2^{ZYG11B}	0.70	$6.91*10^4±5.52*10^3$	14.47	$-1.129*10^4±558.8$	-15.6
SARS-2 ORF10 N4 vs CUL2^{ZYG11B}	0.93	$1.93*10^5±2.99*10^4$	5.18	-7679±304.1	-1.48
SARS-2 ORF10 N5 vs CUL2^{ZYG11B}	0.94	$3.40*10^5±1.27*10^5$	2.94	-6244±434.6	4.43
SARS-2 ORF10 N5-G1A vs CUL2^{ZYG11B}	0.76	$8.09*10^4±2.98*10^4$	12.36	-6979±1337	-0.87
SARS-2 ORF10 N5-G1S vs CUL2^{ZYG11B}	0.65	$7.59*10^4±5.24*10^3$	13.17	$-1.053*10^4±677.9$	-12.90

SARS-2 ORF10 N5-G1P vs CUL2^{ZYG11B}	ND	ND	ND	ND	ND
SARS-2 ORF10 N5-Y2A vs CUL2^{ZYG11B}	0.91	$3.97*10^4±4.22*10^3$	25.18	-7896±620.3	-5.35
SARS-2 ORF10 N5-Y2P vs CUL2^{ZYG11B}	ND	ND	ND	ND	ND
SARS-2 ORF10 N5-Y2F vs CUL2^{ZYG11B}	0.76	$2.11*10^5±1.36*10^4$	4.73	-7523±133	-0.78
SARS-2 ORF10 N5-I3A vs CUL2^{ZYG11B}	0.77	$1.78*10^5±3.4*10^4$	5.61	$-1.067*10^4±614.9$	-11.60
SARS-2 ORF10 N5-I3F vs CUL2^{ZYG11B}	0.68	$1.14*10^6±3.06*10^5$	0.87	$-1.094*10^4±346.8$	-8.86
SARS-2 ORF10 N5-N4A vs CUL2^{ZYG11B}	0.60	$1.69*10^6±1.61*10^5$	0.59	-9756±90.10	-4.11
SNX11-degron-5 vs CUL2^{ZYG11B}	1.27	$2.48*10^6±5.28*10^5$	0.40	-8730±155.0	0.0757
SNX11-degron-5 vs SARS-2 ORF10 N5/CUL2^{ZYG11B}	0.89	$1.91*10^5±5.05*10^4$	5.23	-5113±355.3	7.08

N, stoichiometry

Ka, affinity constant; standard deviation did not exceed ±10%.

Kd, dissociation constant; calculated as 1/Ka.

ΔH, enthalpy variation; standard deviation did not exceed ±10%.

ΔS, entropy variation.

ND, not determined

Table S5. Various ORF10 synthetic peptides used in this study

Peptide #	Peptide name	Sequence (N' to C')
1	SARS-2 ORF10 N2	GY
2	SARS-2 ORF10 N3	GYI
3	SARS-2 ORF10 N4	GYIN
4	SARS-2 ORF10 N5	GYINV
5	SARS-2 ORF10 N7	GYINVFA
6	SARS-2 ORF10 N5-G1A	AYINV
7	SARS-2 ORF10 N5-G1S	SYINV
8	SARS-2 ORF10 N5-G1P	PYINV
9	SARS-2 ORF10 N5-Y2A	GAINV
10	SARS-2 ORF10 N5-Y2P	GPINV
11	SARS-2 ORF10 N5-Y2F	GFINV
12	SARS-2 ORF10 N5-I3A	GYANV
13	SARS-2 ORF10 N5-I3F	GYFNV
14	SARS-2 ORF10 N5-N4A	GYIAV
15	SNX11-degron-5	GFWCR
